# Abdominal obesity and colorectal cancer risk: systematic review and meta-analysis of prospective studies

**DOI:** 10.1042/BSR20170945

**Published:** 2017-12-12

**Authors:** Yunlong Dong, Jiao Zhou, Yun Zhu, Linhai Luo, Tao He, Hong Hu, Hao Liu, Yingliang Zhang, Dan Luo, Shuanglan Xu, Lifen Xu, Jianping Liu, Jun Zhang, Zhaowei Teng

**Affiliations:** 1 Department of General Surgery, The People’s Hospital of Yuxi City, The 6th Affiliated Hospital of Kunming Medical University, Yuxi 653100, China; 2 Department of Gastroenterology, The First Affiliated Hospital of Kunming Medical University, Kunming 650032, China; 3 Department of Nephrology, The People’s Hospital of Yuxi City, The 6th Affiliated Hospital of Kunming Medical University, Yuxi 653100, China; 4 Department of General Surgery, The First Affiliated Hospital of Dali Medical University, Dali 671000, China; 5 Department of Pediatrics, The First Affliated Hospital of Kunming Medical University, Kunming 650032, China; 6 Department of Respiratory, Yan’an Hospital of Kunming City, Kunming 650032, China; 7 Department of Orthopedic Surgery, The People’s Hospital of Yuxi City, The 6th Affiliated Hospital of Kunming Medical University, Yuxi 653100, China; 8 Department of Science and Education, The People’s Hospital of Yuxi City, The 6th Affiliated Hospital of Kunming Medical University, Yuxi 653100, China

**Keywords:** abdominal obesity, central obesity, colorectal cancer, waist circumference, waist to hip ratio

## Abstract

The association between abdominal obesity (as measured by waist circumference (WC) and waist-to-hip ratio (WHR)) and colorectal cancer (CRC) has not been fully quantified, and the magnitude of CRC risk associated with abdominal obesity is still unclear. A meta-analysis of prospective studies was performed to elucidate the CRC risk associated with abdominal obesity. Pubmed and Embase were searched for studies assessing the association between abdominal obesity and CRC risk. Relative risks (RRs) with 95% confidence intervals (95% CIs) were pooled using random-effects model of meta-analysis. Nineteen prospective cohort studies from eighteen publications were included in this meta-analysis. A total of 12,837 CRC cases were identified among 1,343,560 participants. Greater WC and WHR were significantly associated with increased risk of total colorectal cancer (WC: RR 1.42, 95% CI 1.30, 1.55; WHR: RR 1.39, 95% CI 1.25, 1.53), colon cancer (WC: RR 1.53, 95% CI 1.36, 1.72; WHR: 1.39, 95% CI 1.18, 1.63), and rectal cancer (WC: RR 1.20, 95% CI 1.03, 1.39; WHR: RR 1.22, 95% CI 1.05, 1.42). Subgroup analyses further identified the robustness of the association above. No obvious risk of publication bias was observed. In summary, abdominal obesity may play an important role in the development of CRC.

## Introduction

Colorectal cancer (CRC) is a major public health concern, as it is one of the leading causes of cancer deaths in the Western world [1]. Although the high incidence rate of CRC is observed in developed countries, its incidence rate has been rapidly increasing in developing countries over the last few decades [2]. To explore the effective tools for the prevention of CRC, great investment has been made to gain new insight into how environmental factors influence the development of CRC. Several environmental risk factors, such as smoking, obesity, a high-fat/low-fiber diet or physical inactivity, have been suggested for CRC development [3–5].

The prevalence of overweight and obesity is increasing dramatically in most parts of the world, and can lead to obesity-related cancers, including postmenopausal breast cancer, colorectal, endometrial, esophagus, kidney, lung, pancreatic, thyroid, and gallbladder cancers [6–14]. However, the association between obesity and CRC is controversial. In contrast with general obesity, body fat distribution—particularly abdominal obesity—appears to play a role in the development of CRC [15,16]. This positive association of WC or WHR with CRC remained even after adjustment for body mass index (BMI) [15,17,18].

The risk of CRC in obese individuals, especially those with higher abdominal obesity has not been fully quantified, and it is also unclear whether abdominal obesity is an independent risk factor of CRC. Therefore, a comprehensive systematic review and meta-analysis of prospective studies was performed to estimate the risk of CRC associated with abdominal obesity.

## Materials and methods

### Search strategy

The present study was planned, conducted, and reported in adherence to the “Meta-Analysis of Observational Studies in Epidemiology (MOOSE)” guidelines [19]. We systematically searched Pubmed and Embase (from their commencements to April 28, 2017) for studies of the association between abdominal obesity and colorectal cancer risk. We used the following search terms: (obesity OR adiposity OR body size OR body fat distribution OR anthropometric OR anthropometry OR waist-to-hip ratio OR WHR OR waist circumference OR WC) AND (colorectal cancer OR colorectal neoplasm OR colon cancer OR colon neoplasm OR rectal cancer OR rectal neoplasm) AND (cohort OR prospective OR follow-up). The search strategy had no language, publication date, or publication type restriction. In addition, the reference lists of relevant reviews or included articles were also searched to find other eligible studies.

### Study selection

Studies were included if they met all of the following criteria: (a) prospective cohort studies; (b) the exposure of interest was abdominal obesity (measured using WC and/or WHR); (c) the outcome of interest was colorectal cancer; (d) risk estimates of colorectal cancer associated with abdominal obesity were available, such as relative risks (RRs) or hazard ratios (HRs) with 95% confidence intervals (CIs); and (e) the risk estimates were adjusted for other confounding factors. Studies were excluded if they focused on colorectal mortality or recurrence.

### Data extraction and quality assessment

Using a standardized extraction form, the following data were extracted from each study: the first author’s last name, publication year, country, study period, age range, sex, number of cases, number of participants, data collection, measures of abdominal adiposity, cancer sites, most fully adjusted risk estimates with their corresponding 95% CIs for each category of abdominal adiposity measures, and adjustment for potential confounding factors. Risk estimates reported by gender were extracted separately. Newcastle–Ottawa Scale (NOS) was used to assess the quality of included studies [20]. According to the quality criteria, four points were awarded for the selection of the study groups (representativeness, selection of non-exposed cohort, ascertainment of exposure, and no disease at start of study), two points for the comparability of groups, and three points for the assessment of outcomes (assessment of outcome, length of follow-up, and adequacy of follow-up). Quality was assigned as excellent with seven to nine stars, good with four to six stars, and suboptimal with zero to three stars. Two investigators (Y.L.D. and Z.W.T.) participated in literature search, study selection, data extraction, and quality assessment independently. Disagreements were resolved by discussion.

### Statistical analysis

HR was directly considered as RR across the present study. The pooled RRs with 95% CIs were calculated using a random-effects model with the method of DerSimonian and Laird [21]. Because WC and WHR are different among races and studies, and have no unified criteria around the world, we combined the different outcomes between the smallest WC or WHR quantile and the largest WC or WHR quantile in studies using relative risks in meta-analysis of random effects. The degree of heterogeneity in the relationship between measures of abdominal obesity and colorectal cancer across studies was assessed using *Q* and *I*^2^ statistics. For the *Q* statistic, *P*<0.1 was considered statistically significant; and for the *I*^2^ statistic, *I*^2^ more than 40% indicated substantial heterogeneity across included studies, and metaregression was further utilized to find the source of heterogeneity. Both Begg’s and Egger’s tests were performed to investigate potential publication bias [22,23]. To explore potential sources of heterogeneity, subgroup analyses were performed by anatomical subsite, geographic region, sex, and data collection. To evaluate the effect of an individual article on the overall pooled results, a sensitivity analysis was conducted by omitting each article from the overall analysis in every turn. All statistical analyses were performed using STATA, version 12.0 (STATA Corp., College Station, Texas, U.S.A.). A two-tailed *P*-value of less than 0.05 was considered to be statistically significant.

## Results

### Literature search

**Figure 1:**
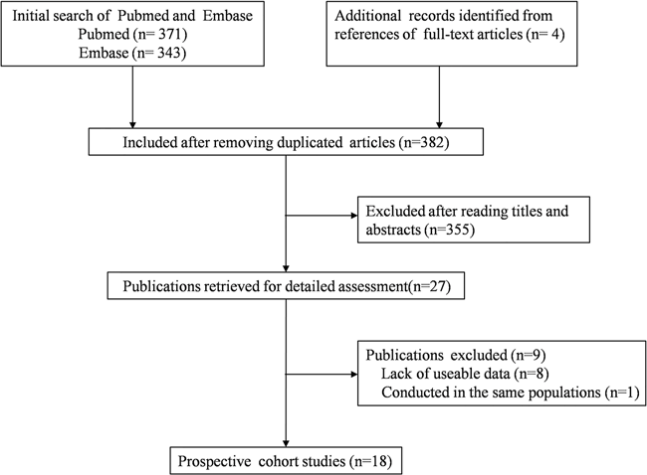
Flow chart of study selection in the meta-analysis .

A total of 714 articles were initially identified from Pubmed and Embase databases. After removing the duplicated articles, 382 studies were included for further assessment. Of these articles, 355 studies were excluded after reading the titles and the abstracts. After full-text review of the remaining 27 articles, 18 studies [15,17,18,24–38] were included in the final meta-analysis (Figure 1). Four [35–38] of the eighteen articles were identified from references of three full-text articles.

### Study characteristics

The general characteristics of the included studies are presented in Table 1. The included studies which were published from 1994 to 2015 were all prospective cohort studies. There were 12,837 colorectal cancer cases among 1,343,560 participants. Of these 18 studies, 1 was conducted in Asia [26], 11 in the U.S.A. [15,17,24,25,27,28,33,34,36–38], 3 in Europe [18,29,35], and 3 in Australia [30–32]. Among the studies, 3 concerned about men [15,29,32], 6 about women [17,24,27,31,34,37], and 9 about both genders [18,25,26,28,30,33,35,36,38]. Nine studies relied on self-reported data [15,17,25,27–29,34,35,37], and 10 studies relied on measured data [18,24,26,30–33,35,36,38]. Individual studies adjusted for a wide range of potential confounding factors, such as age, physical activity, and alcohol intake. The details of quality assessment according to the nine-star NOS are presented in the online Supplementary Table S1. All studies were given scores of ≥6.

**Table 1:** Baseline characteristics of the included studies

Study	Study period	Age range	Sex	Cases/Cohort size	Data collection	Cancer sites	Measure of adiposity	Categories, highest vs. lowest (measurement unit)	Adjusted RR (95% CI)	Adjustments
Moore (ages 30–54 y) (2004) U.S.A. [33]	1948–1999	30–54	M/F	157/3764	Measured	CC	WC	Men: <83.8 cm vs. ≥101.6 cm	CC: 2.9 (1.2–6.7)	BMI, sex, education, age, height, alcohol intake, cigarettes per day, physical activity
									CC: men 3.3 (0.91–12.3); women 2.3 (0.74–7.0)	
								Women: <81.3 cm vs. ≥99.1 cm	Proximal CC: 3.0 (1.0–8.6)	
									Distal CC: 2.5 (0.59–10.6)	
Moore (ages 55–79 y) (2004) U.S.A. [33]	1948–1999	55–79	M/F	149/3802	Measured	CC	WC	Men: <83.8 cm vs. ≥101.6 cm	CC: 2.4 (1.0–5.6)	BMI, education, age, height, alcohol intake, cigarettes per day, physical activity
									CC: men 3.0 (0.86–10.3); women 2.1 (0.63–6.7)	
								Women: <81.3 cm vs. ≥99.1 cm	Proximal CC: 2.4 (0.78–7.1)	
									Distal CC: 2.4 (0.62–9.2)	
Larsson (2006) Sweden [29]	1998–2005	45–79	M	496/45906	Self-report	CC/RC/CRC	WC	Men: <88 cm vs. ≥104 cm	CRC: men 1.29 (0.90–1.85)	Age, education, family history of colorectal cancer, history of diabetes, smoking, aspirin use, leisure-time physical activity, height
									CC: men 1.44 (0.93–2.24)	
									Proximal CC: men 1.66 (0.84–3.27)	
									Distal CC: men 1.62 (0.80–3.27)	
									RC: men 1.24 (0.68–2.25)	
Pischon (2006) Europe [18]	1992–2000	25–70	M/F	1570/368277	Measured	CC/RC	WC	Men: <86.0 cm vs. ≥103.0 cm	CC: men 1.39 (1.01–1.93); women 1.48 (1.08–2.03)	Age, center and age at recruitment, smoking status, education, alcohol intake, physical activity, fiber intake, consumption of red and processed meat, fish and shellfish, fruits and vegetables, height
								Women: <70.2 cm vs. ≥89.0 cm	RC: men 1.27 (0.84–1.91); women 1.23 (0.81–1.86)	
							WHR	Men: <0.887 vs. ≥0.990	CC: men 1.51 (1.06–2.15); women 1.52 (1.12–2.05)	
								Women: <0.734 vs. ≥0.846	RC: men 1.93 (1.19–3.13); women 1.20 (0.81–1.79)	
Maclnnis (2005) Australia [31]	1990–2003	27–75	F	212/24072	Measured	CC	WC	Women: <80 cm vs. ≥88 cm	CC: women 1.4 (1.0–1.9)	Country of birth, highest level of education, hormone replacement therapy use
							WHR	Women: <0.75 vs. ≥0.80	CC: women 1.7 (1.1–2.4)	
Giovannucci (1995) U.S.A. [15]	1987–1992	40–75	M	203/47723	Self-report	CC	WC	Men: <35 in vs. ≥43 in	CC: men 2.56 (1.33–4.96)	Age, history of endoscopic screening, previous polyp diagnosis, parental history of colorectal cancer, pack-years of smoking, physical activity, aspirin use, and intake of folate, methione, alcohol, dietary fiber, total energy, and red meat
							WHR	Men: <0.90 vs. ≥0.99	CC: men 3.41 (1.52–7.66)	
Martinez (1997) U.S.A. [17]	1986–1992	30–55	F	212/67802	Self-report	CC	WC	Women: ≤27.5 in vs. >34 in	CC: women 1.48 (0.89–2.46)	ND
									Distal CC: women 1.47 (0.71–3.06)	
							WHR	Women: <0.728 vs. >0.833	CC: women 1.48 (0.88–2.49)	Age, cigarette smoking, family history of colorectal cancer, leisure-time physical activity, postmenopausal hormone use, aspirin use, intake of red meat, and alcohol consumption
									Proximal CC: women 1.66 (0.69–3.99)	
									Distal CC: women 1.79 (0.82–3.90)	
Bostick (1994) U.S.A. [34]	1986–1990	55–69	F	212/35215	Self-report	CC	WHR	Women: <0.764 vs. >0.906	CC: women 1.25 (0.83–1.88)	Age, total energy intake, height, parity, total vitamin E intake, a total vitamin E by age interaction term, and vitamin A supplement intake
Maclnnis (2004) Australia [32]	1991–2002	27–75	M	153/16556	Measured	CC	WC	Men: <87.0 cm vs. >99.3 cm	CC: men 2.1 (1.3–3.5)	Age at attendance, country of birth, highest level of education
							WHR	Men: <0.88 vs. >0.96	CC: men 2.1 (1.3–3.4)	
Wang (2008) U.S.A. [28]	1997–2005	≥45	M/F	953/95151	Self-report	CC/RC/CRC	WC	Men: <95 cm vs. ≥120 cm	CRC: men 1.68 (1.12–2.53); women 1.75 (1.20–2.54)	Height, education, physical activity, smoking, alcohol intake, NSAID use, multivitamin use, and history of colorectal endoscopy (women+HRT use)
								Women: <85 cm vs. ≥110 cm	CC: men 2.05 (1.29–3.25); women 1.54 (1.00–2.37)	
									RC: men 1.02 (0.43–2.42); women 2.65 (1.23–5.71)	
Oxentenko (2010) U.S.A. [27]	1986–2005	55–69	F	1464/36941	Self-report	CRC	WC	Women: ≤77.15 cm vs. ≥96.53 cm	CRC: women 1.32 (1.11–1.56)	Age at baseline, age at menopause, exogenous estrogen use, oral contraceptive use, smoking status, cigarette pack-years, physical activity level, self-reported diabetes mellitus, and intake of total energy, total fat, red meat, fruits and vegetables, calcium, folate, vitamin E and alcohol
							WHR	Women: ≤0.78 vs. ≥0.90	CRC: women 1.28 (1.08–1.50)	
Li (2013) China [26]	1997–2009	40–74	M/F	935/134255	Measured	CC/RC/CRC	WC	Men: <78 cm vs. ≥92 cm	CRC: men 1.38 (0.97–1.97); women 1.26 (0.93–1.72)	Age at baseline, education, income, pack-years of cigarette use, tea consumption, alcohol consumption, physical activity, family history of colorectal cancer and intakes of total energy, red meat, fruits and vegetables
								Women: <70 cm vs. ≥85 cm	CC: men 2.00 (1.21–3.29); women 1.34 (0.89–2.00)	
									RC: men 0.88 (0.52–1.49); women 1.17 (0.73–1.88)	
							WHR	Men: <0.85 vs. ≥0.95	CRC: men 1.65 (1.12–2.41); women 1.01 (0.79–1.31)	
								Women: <0.77 vs. ≥0.85	CC: men 1.97 (1.19–3.24); women 0.96 (0.69–1.34)	
									RC: men 1.24 (0.69–2.26); women 1.11 (0.74–1.66)	
Keimling (2013) U.S.A. [25]	1995–2006	50–71	M/F	2869/203177	Self-report	CC/RC	WC	Men: <89.5 cm vs. ≥106.5 cm	CC: men 1.45 (1.16–1.82); women 0.90 (0.63–1.27)	Age, education, race/ethnicity, smoking status, marital status, physical activity, NSAID use, family history of colorectal cancer, diabetes status, dietary intakes of total energy, fiber, folate, calcium, red meat, fruits and vegetables, alcohol, HRT, height (WC+hip circumference)
								Women: <73.6 cm vs. ≥94.5 cm	Proximal CC: women 0.86 (0.56–1.32)	
									Distal CC: women 1.00 (0.54–1.84)	
									RC: men 0.97 (0.67–1.38); women 1.01 (0.53–1.94)	
							WHR	Men: <0.898 vs. ≥1.000	CC: men 1.29 (1.10–1.52); women 0.90 (0.70–1.15)	
								Women: <0.746 vs. ≥0.877	Proximal CC: women 0.73 (0.53–1.01)	
									Distal CC: women 1.23 (0.80–1.90)	
									RC: men 1.08 (0.82–1.43); women 1.13 (0.69–1.86)	
Kabat (2015) U.S.A. [24]	1993–2013	50–79	F	1908/143901	Measured	CRC	WC	ND	CRC: women 1.90 (1.61–2.25)	Age, alcohol, smoking, hormone therapy, MET-hours/week, aspirin intake, diabetes, family history of colorectal cancer in a first-degree relative, education, ethnicity, treatment allocation
							WHR	ND	CRC: women 1.65 (1.40–1.93)	
Park (2011) U.K. [35]	1993–2006	40–79	M/F	357/24244	Measured self-report	CRC	WC	Men: <88.0 cm vs. ≥103.3 cm	Measured CRC: men 0.86 (0.55–1.36); women 1.65 (0.97–2.86)	Age, sex, smoking, alcohol, education, exercise, family history of CRC, energy intake, folate, fiber, total meat and processed meat, intakes, height
								Women: <73.0 cm vs. ≥90.5 cm	Self-report CRC: men 0.95 (0.54–1.64); women 1.42 (0.85–2.35)	
							WHR	Men: <0.883 vs. ≥0.979	Measured CRC: men 1.34 (0.79–2.25); women 2.07 (1.17–3.67)	
								Women: <0.739 vs. ≥0.844	Self-report CRC: men 1.79 (0.88–3.62); women 1.26 (0.75–2.13)	
Folsom (2000) U.S.A. [37]	1986–1996	55–69	F	462/31702	Self-report	CC	WC	Women: <74.3 cm vs. ≥96.0 cm	CC: women 1.6 (1.2–2.2)	Age, educational level, physical activity, alcohol intake, smoking status, pack-years of cigarette smoking, age of first live birth, estrogen use, vitamin use, and energy, whole grain, fruit and vegetable, fish, and red meat intake and keys score
							WHR	Women: <0.762 vs. ≥0.901	CC: women 1.2 (0.9–1.7)	
Schoen (1999) U.S.A. [38]	1989–1996	≥65	M/F	102/5849	Measured	CRC	WC	Men: 69–91 cm vs. 104.1–145.5 cm	CRC: 2.2 (1.2–4.1)	Age, sex, and physical activity
								Women: 32.5–82 cm vs. 101.2–167 cm		
							WHR	Men: 0.61–0.93 vs. 1.01–2.33	CRC: 2.6 (1.4–4.8)	
								Women: 0.61–0.83 vs. 0.961–2.06		
MacInnis (2006) Australia [30]	1990–2003	27–75	M/F	229/41114	Measured	RC	WC	Men: <94 cm vs. ≥102 cm	RC: 1.4 (1.0–1.9)	Age as the time axis, sex, and country of birth
								Women: <80 cm vs. ≥88 cm	RC: men 1.4 (0.9–2.2); women 1.4 (0.8–2.2)	
							WHR	Men: <0.90 vs. ≥0.95	RC: 1.3 (0.9–1.8)	
								Women: <0.75 vs. ≥0.80	RC: men 1.2 (0.8–1.8); women 1.4 (0.8–2.4)	
Ahmed (2006) U.S.A. [36]	1987–2000	45–64	M/F	194/14109	Measured	CRC	WC	Men: <102 cm vs. ≥102 cm	CRC: 1.40 (1.0–1.9)	Family history of colorectal cancer, physical activity, nonsteroidal anti-inflammatory drug use, aspirin use, pack-years of cigarette use, and grams of alcohol per week (women+HRT use)
								Women: <88 cm vs. ≥88 cm		

Abbreviations: 95% CI, 95% confidence interval; BMI, body mass index; CC, colon cancer; CRC, colorectal cancer; F, female; HRT, hormone replacement therapy; M, male; ND, no data; RC, rectal cancer; RR, relative risk; WC, waist circumference; WHR, waist-to-hip ratio.

### WC and colorectal cancer

**Figure 2:**
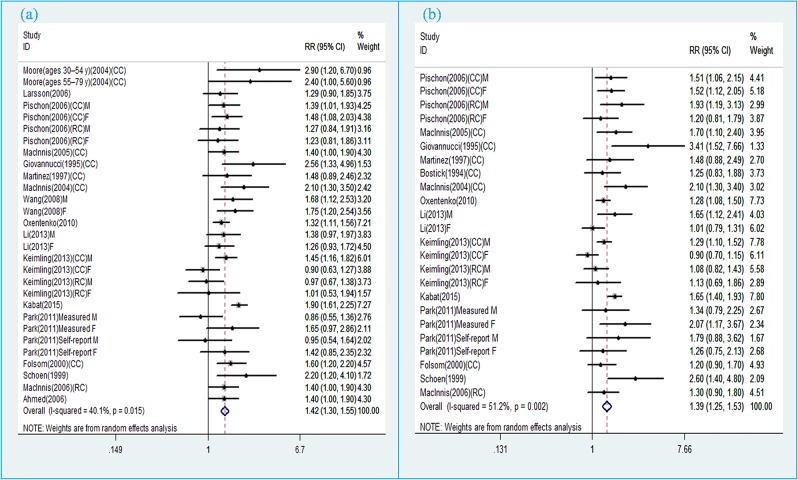
(**a**) Pooled relative risk of CRC associated with waist circumference; (**b**) Pooled relative risk of CRC associated with waist-to-hip ratio.

**Figure 3:**
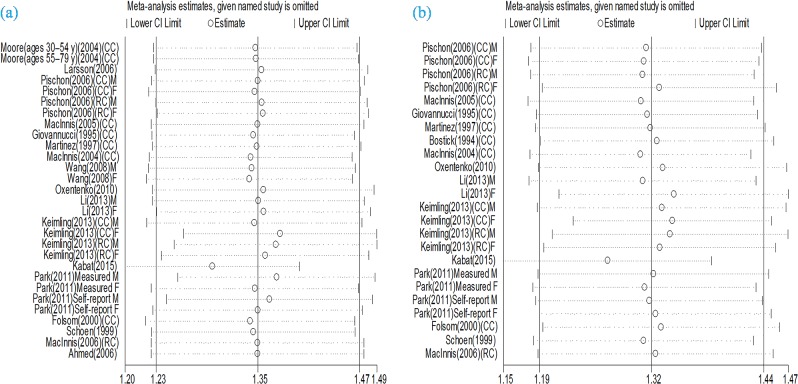
(**a**) Sensitivity analysis of CRC associated with waist circumference; (**b**) Sensitivity analysis of CRC associated with waist-to-hip ratio.

**Figure 4:**
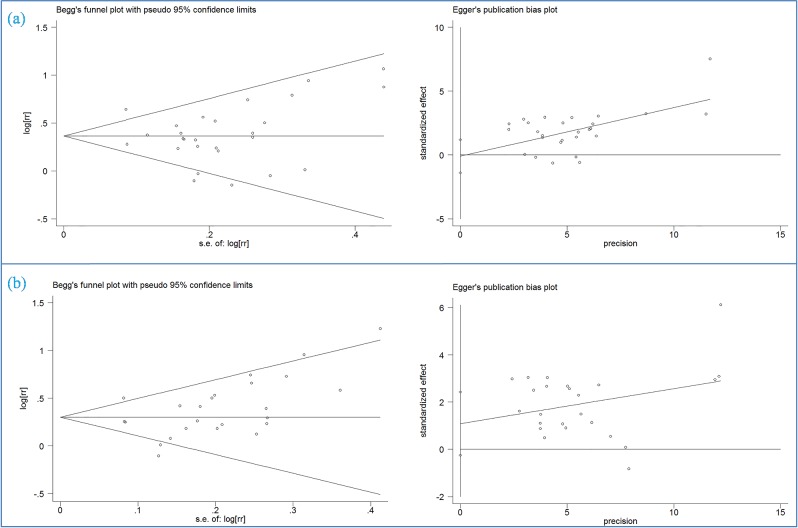
(**a**) Both Begg’s rank correlation test and Egger’s linear regression test of CRC associated with waist circumference; (**b**) Both Begg’s rank correlation test and Egger’s linear regression test of CRC associated with waist-to-hip ratio.

Eighteen prospective cohort studies [15,17,18,24–33,35–38] were included in the analysis of WC and risk of colorectal cancer incidence. Comparison of the highest category of WC with the lowest category revealed significant associations between greater WC and increased risk of total colorectal cancer (RR 1.42, 95% CI 1.30, 1.55), colon cancer (RR 1.53, 95% CI 1.36, 1.72), and rectal cancer (RR 1.20, 95% CI 1.03, 1.39) (Figure 2a). The evidence of moderate heterogeneity were observed for total colorectal cancer (*I*^2^ = 40.1%, *P*=0.015), while low heterogeneity was observed for colon cancer (*I*^2^ = 24.6%, *P*=0.176). On the other hand, no evidence of heterogeneity for rectal cancer (*I*^2^ = 0.0%, *P*=0.518). Subgroup analyses further identified the robustness of the association between abdominal obesity and CRC risk (Table 2). Stratifying by sex, the pooled RRs of CRC from male and female CRC studies for the highest vs. lowest categories of WC level were 1.38 (95% CI, 1.19–1.59) and 1.44 (95% CI, 1.28–1.61) respectively. Stratifying by geographic region, the pooled RRs of CRC for the highest vs. lowest categories of WC level were 1.31 (95% CI, 1.04–1.65) for studies conducted in Asia, 1.50 (95% CI, 1.30–1.74) for studies in the United States, 1.29 (95% CI, 1.13–1.48) for studies in Europe, and 1.51 (95% CI, 1.22–1.87) for studies in Australia. When we stratified the analysis by measured and self-reported CRC data, the pooled RRs of measured and self-reported CRC data were 1.48 (95% CI, 1.32–1.67) and 1.35 (95% CI, 1.18–1.53) for the highest vs. lowest categories of WC levels respectively. Metaregression analysis showed that geographic region was the source of heterogeneity. The sensitivity analyses omitting one study at a time and calculating the combined RRs for the remaining studies showed that the combined RRs were not substantially affected by any single study (Figure 3a). The Begg’s and Egger’s tests indicated no evidence of publication bias among the studies [Begg, *P* > |*z*| = 0.431; Egger, *P* = 0.862, 95% CI, 1.402–1.181] (Figure 4a).

**Table 2:** Subgroup analyses of CRC risk associated with abdominal obesity

Subgroup	WC			WHR		
	Studies	RR (95% CI)	Heterogeneity (*P*-value, *I*^2^)	Studies	RR (95% CI)	Heterogeneity (*P*-value, *I*^2^)
Anatomical subsite						
Colorectal cancer	18	1.42 (1.30–1.55)	0.015, 40.1%	14	1.39 (1.25–1.53)	0.002, 51.2%
Colon cancer	12	1.53 (1.36–1.72)	0.176, 24.6%	9	1.39 (1.18–1.63)	0.004, 60.2%
Rectal cancer	6	1.20 (1.03–1.39)	0.518, 0.0%	4	1.22 (1.05–1.42)	0.591, 0.0%
Geographic region						
Asia	1	1.31 (1.04–1.65)	0.704, 0.0%	1	1.26 (0.78–2.04)	0.036, 77.2%
U.S.A.	11	1.50 (1.30–1.74)	0.002, 58.6%	8	1.32 (1.13–1.54)	0.001, 66.3%
Europe	3	1.29 (1.13–1.48)	0.628, 0.0%	2	1.51 (1.29–1.76)	0.741, 0.0%
Australia	3	1.51 (1.22–1.87)	0.345, 6.0%	3	1.61 (1.23–2.10)	0.260, 25.8%
Sex						
Colorectal cancer						
Men	11	1.38 (1.19–1.59)	0.088, 36.0%	7	1.47 (1.25–1.73)	0.083, 41.2%
Women	13	1.44 (1.28–1.61)	0.075, 36.1%	11	1.30 (1.15–1.48)	0.012, 52.0%
Colon cancer						
Men	9	1.67 (1.43–1.94)	0.366, 8.3%	5	1.71 (1.29–2.27)	0.039, 60.3%
Women	9	1.39 (1.21–1.60)	0.392, 5.2%	7	1.22 (1.01–1.48)	0.044, 53.6%
Rectal cancer						
Men	6	1.12 (0.92–1.36)	0.717, 0.0%	4	1.27 (0.99–1.62)	0.242, 28.4%
Women	5	1.31 (1.03–1.67)	0.376, 5.4%	4	1.19 (0.95–1.49)	0.920, 0.0%
Data collection						
Measured	11	1.48 (1.32–1.67)	0.069, 36.9%	8	1.53 (1.34–1.75)	0.056, 41.8%
Self-report	8	1.35 (1.18–1.53)	0.086, 37.2%	7	1.22 (1.08–1.38)	0.122, 34.6%

Abbreviations: CI, confidence interval; RR, relative risk; WC, waist circumference; WHR, waist-to-hip ratio.

### WHR and colorectal cancer

Fourteen prospective cohort studies [15,17,18,24–27,30–32,34,35,37,38] were included in the analysis of WHR and risk of colorectal cancer incidence. Comparison of the highest category of WHR with the lowest category revealed significant associations between higher WHR and increased risk of total colorectal cancer (RR 1.39, 95% CI 1.25, 1.53), colon cancer (RR 1.39, 95% CI 1.18, 1.63), and rectal cancer (RR 1.22, 95% CI 1.05, 1.42) (Figure 2b). The evidence of high heterogeneity were observed for total colorectal cancer (*I*^2^ = 51.2%, *P*=0.002), as well as for colon cancer (*I*^2^ = 60.2%, *P* = 0.004), while no heterogeneity was observed for rectal cancer (*I*^2^ = 0.0%, *P*=0.591). Subgroup analyses further identified the robustness of the association between abdominal obesity and CRC risk (Table 2). Stratifying by sex, the pooled RRs of CRC from male and female CRC studies for the highest vs. lowest categories of WHR level were 1.47 (95% CI, 1.25–1.73) and 1.30 (95% CI, 1.15–1.48) respectively. Stratifying by geographic region, the pooled RRs of CRC for the highest vs. lowest categories of WHR level were 1.26 (95% CI, 0.78–2.04) for studies conducted in Asia, 1.32 (95% CI, 1.13–1.54) for studies in the United States, 1.51 (95% CI, 1.29–1.76) for studies in Europe, and 1.61 (95% CI, 1.23–2.10) for studies in Australia. When we stratified the analysis by measured and self-reported CRC data, the pooled RRs of measured and self-reported CRC data were 1.53 (95% CI, 1.34–1.75) and 1.22 (95% CI, 1.08–1.38) for the highest vs. lowest categories of WHR levels respectively. Metaregression analysis showed that geographic region and sex were possible sources of heterogeneity. The sensitivity analyses omitting one study at a time and calculating the combined RRs for the remaining studies showed the combined RRs were not substantially affected by any single study (Figure 3b). The Begg’s and Egger’s test indicated no evidence of publication bias among the studies [Begg, *P* > |*z*| = 0.009; Egger, *P*=0.106; 95% CI, 0.248–2.419] (Figure 4b).

## Discussion

This systematic review and meta-analysis aimed to examine the association between abdominal obesity and risk of total colorectal cancer, colon cancer, and rectal cancer, which was the first meta-analysis on this subject. We found evidence of an increased risk of total colorectal cancer, colon cancer, and rectal cancer with greater WC and WHR.

There was obvious heterogeneity across those included studies. In some subgroup analysis, the heterogeneity declines (Table 2). However, the heterogeneity of other subgroup analysis remains high, which suggested that much of the heterogeneity in the meta-analysis was unable to be unexplained by subgroup analyses. In the metaregression analyses, the pooled risk estimates were similar when studies were stratified by those factors that were identified as possible sources of heterogeneity, and almost all the pooled risk estimates were statistically significant (Table 2). Thus, the heterogeneity had little influence on the overall evidence for the association between abdominal obesity and colorectal cancer.

Strengths of the study are as follows: (a) To capture all relevant information, studies were included after a comprehensive, systematic search of the literature by a multidisciplinary team including specialists in gastrointestinal endoscopy, gastroenterology, and clinical epidemiology and using a broad search strategy. (b) Most of the included studies adjusted for nearly all the important covariates including age, education, family history of colorectal cancer, physical activity, alcohol intake, smoking etc. (c) The present meta-analysis is based on prospective studies, so we have effectively avoided recall and selection bias.

Obesity is considered one of the important risk factors for many types of cancer, especially for CRC. However, the mechanisms that might underlie the association between excess weight and CRC remain unclear. Several mechanisms have been proposed to explain how general and central obesity enhances colorectal neoplasm risk. Previous studies have demonstrated that the fat itself can also influence CRC risk [39]. Adipocytes and preadipocytes could promote proliferation of CRC cells [40]. Fatty acid synthase overexpression has been shown to be associated with CRC phenotype [41]. Adipokines such as adiponectin, leptin are also associated with the risk of CRC. Adiponectin as an insulin-sensitizing agent and a negative regulator of angiogenesis is secreted mainly from visceral adipose tissue, which could inhibit CRC growth in animal models, and its circulating concentrations were associated with CRC risk in clinical trials [42]. Leptin could also favor CRC growth *in vivo* and *in vitro* experiment as a pleiotropic hormone being mitogenic, anti-apoptotic, pro-angiogenic, and proinflammatory in various cellular systems [43]. The relationship between circulating leptin concentrations and CRC risk has been demonstrated [44]. In addition, obesity, particularly abdominal obesity, is linked to insulin resistance, to hyperinsulinemia, and to the development of Type 2 diabetes [45,46]. IGF binding protein-1 (IGFBP-1) concentrations decrease with increasing adiposity [47], which may lead to elevated concentrations of free and bioavailable insulin-like growth factor-1 (IGF-1) [48]. The involvement of insulin and the subsequent up-regulated level of IGF-1 in colorectal carcinogenesis have been supported by experimental and clinical studies [49].

Available epidemiologic evidence suggests that abdominal obesity (as reflected by high WC and WHR) may be more predictive of colon cancer risk than overall obesity (high BMI) [50–53]. This positive association of WC or WHR with CRC remained even after adjustment for BMI [50–52]. The results indicated that higher WC and WHR levels were positively associated with CRC risk. Analyses stratified by the anatomical subsite suggested that both of higher BMI and WC levels caused an increasing risk for colon cancer and rectal cancer. When the analysis was stratified by sex, the results showed that higher WC and WHR levels were significantly positively associated with colorectal and colon cancer risk in both men and women. Stratifying by geographic region, the results revealed that higher BMI and WC levels were positively associated with CRC risk in the United States, Europe, or Australia. In addition, when the analysis was stratified by data collection, the result showed that there was an increased risk of CRC development associated with higher BMI and WC levels for both measured and self-reported data.

However, several limitations in this meta-analysis should be considered. First, most of the included studies did not provide the risk estimates controlling for weight change during follow-up, and they could not exclude the impact of weight change during follow-up on the association between abdominal obesity and CRC. Second, although individual studies have considered a wide range of potential confounders in their analyses, we cannot fully exclude unknown or residual confounding factors which may have influence on our findings. Third, although our analysis indicate that both higher WC and WHR increase the risk of colorectal cancer, colon cancer, and rectal cancer, few studies have conducted further adjustments between abdominal obesity measurement and BMI to try to clarify their respective roles. Finally, as with any meta-analysis, publication bias is a matter of concern, because small studies with null results tend not to be published. Although there was no evidence of publication bias, we cannot exclude such bias because of low statistical power due to limited number of studies.

## Conclusions

In summary, findings from this meta-analysis of prospective studies provide evidence that abdominal obesity may play an important role in the development of colorectal cancer. This positive association also exists in both men and women, different geographic region, and different anatomical site. Further large prospective studies are necessary to evaluate whether the association between central obesity and CRC is biased by BMI.

## Supporting information

**Figure S1:** (a) Pooled relative risk of CC associated with waist circumference; (b) Pooled relative risk of CC associated with waist to hip ratio; (c) Pooled relative risk of RC associated with waist circumference; (d) Pooled relative risk of RC associated with waist to hip ratio

**Table S1:** Quality assessment according to the nine-star Newcastle-Ottawa Scale (NOS)

**Table S2:** Detailed amounts of waist circumference and waist to hip ratio.

## Abbreviations

BMI: body mass index
CRC: colorectal cancer
IGF-1: insulin-like growth factor-1
NOS: Newcastle–Ottawa Scale
WC: waist circumference
WHR: waist-to-hip ratio
