# Supplementary material for: Abdominal obesity and colorectal cancer risk: systematic review and meta-analysis of prospective studies
Source: Biosci Rep. 2017 Dec 12;37(6):BSR20170945. doi: 10.1042/BSR20170945 (PMC5725611; doi:10.1042/BSR20170945)
Supplement: Supplementary file 1 [file bsr20170945_Supp1.pdf]

Supplementary Materials: Abdominal Obesity and Colorectal Cancer Risk: Systematic Review and Meta-Analysis of Prospective Studies

Yunlong Dong, Jiao Zhou, Yun Zhu, Tao He, Hong Hu, Hao Liu, Dan Luo, Shuanglan Xu, Lifen Xu, Jun Zhang and Zhaowei Teng

Table S1. Quality assessment according to the nine-star Newcastle-Ottawa Scale (NOS)[a].

	Selection				Comparability	Outcome			
Study	Representativeness of Exposed Cohort	Selection of the Non-Exposed Cohort	Ascertainment of Exposure	Demonstration That Outcome of Interest Was Not Present at Start of Study	Comparability of Cohorts on the Basis of the Design or Analysis	Assessment of Outcome	Was Followed Up Long Enough for Outcomes to Occur	Adequacy of Follow Up of Cohorts	Total Stars
Moore(ages 30–54 y)(2004) USA	1	1	1	1	2	1	1	0	8
Moore(ages 55–79 y)(2004) USA	1	1	1	1	2	1	1	0	8
Larsson(2006) Sweden	0	1	1	1	2	1	1	0	7
Pischon(2006) Europe	1	1	1	1	2	1	1	0	8
Maclnnis(2005) Australia	0	1	1	1	2	1	1	0	7
Giovannucci(1995) USA	1	1	1	1	2	1	1	0	8
Martinez(1997) USA	1	1	1	1	2	1	1	0	8
Bostick(1994) USA	0	1	1	1	2	1	1	0	7
Maclnnis(2004) Australia	0	1	1	1	2	1	1	0	7
Wang(2008) USA	1	1	1	1	2	1	1	0	8
Oxentenko(2010) USA	1	1	1	1	2	1	1	0	8
Li(2013) China	0	1	1	1	2	1	1	0	7
Keimling(2013) USA	0	1	1	1	2	1	1	0	7
Kabat(2015) USA	0	1	1	1	2	1	1	0	7
Park(2011) UK	1	1	1	1	2	1	1	0	8
Folsom(2000) USA	0	1	1	1	2	1	1	0	7
Schoen(1999) USA	0	1	1	1	1	1	1	0	6
MacInnis(2006) Australia	0	1	1	1	2	1	1	0	7
Ahmed(2006) USA	1	1	1	1	2	1	1	0	8

[a] A study can be awarded a maximum of one star for each numbered item within the Selection and Outcome categories and a maximum of two stars for Comparability.

Table S2. Detailed amounts of waist circumference and waist to hip ratio.

Study	Measure of Adiposity	Categories(Measurement Unit)	Highest vs. Lowest(Measurement Unit)
Moore(ages 30–54 y)(2004) USA	WC	Men: <83.8cm 83.8-<94.0cm 94.0-<101.6cm ≥101.6cm	Men: <83.8cm vs. ≥101.6cm
		Women: <81.3cm 81.3-<91.4cm 91.4-< 99.1cm ≥ 99.1cm	Women: <81.3cm vs. ≥99.1cm
Moore(ages 55–79 y)(2004) USA	WC	Men: <83.8cm 83.8-<94.0cm 94.0-<101.6cm ≥101.6cm	Men: <83.8cm vs. ≥101.6cm
		Women: <81.3cm 81.3-<91.4cm 91.4-< 99.1cm ≥ 99.1cm	Women: <81.3cm vs. ≥99.1cm
Larsson(2006) Sweden	WC	Men: <88cm 88-92cm 93-97cm 98-103cm ≥104cm	Men: <88cm vs. ≥104cm
Pischon(2006) Europe	WC	Men: <86.0cm 86.0-91.8cm 91.9-96.5cm 96.6-102.9cm ≥103.0cm	Men: <86.0cm vs. ≥103.0cm
		Women: <70.2cm 70.2-75.8cm 75.9-80.9cm 81.0-88.9cm ≥89.0cm	Women: <70.2cm vs. ≥89.0cm
	WHR	Men: <0.887 0.887-0.922 0.923-0.952 0.953-0.989 ≥0.990	Men: <0.887 vs. ≥0.990
		Women: <0.734 0.734-0.768 0.769-0.802 0.803-0.845 ≥0.846	Women: <0.734 vs. ≥0.846
Maclnnis(2005) Australia	WC	Women: <80cm 80-87cm ≥88cm	Women: <80cm vs. ≥88cm
	WHR	Women: <0.75 0.75-0.79 ≥0.80	Women: <0.75 vs. ≥0.80
Giovannucci(1995) USA	WC	Men: <35in 35-36.9in 37-38.9in 39-42.9in ≥43in	Men: <35in vs. ≥43in
	WHR	Men: <0.90 0.90-0.92 0.93-0.94 0.95-0.98 ≥0.99	Men: <0.90 vs. ≥0.99
Martinez(1997) USA	WC	Women: ≤27.5in >34in	Women: ≤27.5in vs. >34in
	WHR	Women: <0.728 0.728-0.758 0.759-0.790 0.791-0.833 >0.833	Women: <0.728 vs. >0.833
Bostick(1994) USA	WHR	Women: <0.764 0.764-0.808 0.809-0.852 0.853-0.906 >0.906	Women: <0.764 vs. >0.906
Maclnnis(2004) Australia	WC	Men: <87.0cm 87.0-92.9cm 93.0-99.3cm >99.3cm	Men: <87.0cm vs. >99.3cm
	WHR	Men: <0.88 0.88-0.92 0.93-0.96 >0.96	Men: <0.88 vs. >0.96
Wang(2008) USA	WC	Men: <95cm 95-<105cm 105-<120cm ≥120cm	Men: <95cm vs. ≥120cm
		Women: <85cm 85-<95cm 95-<110cm ≥110cm	Women: <85cm vs. ≥110cm
Oxentenko(2010) USA	WC	Women: ≤77.15cm 77.16-86.04cm 86.05-96.52cm ≥96.53cm	Women: ≤77.15cm vs. ≥96.53cm

	WHR	Women: ≤0.78 0.79-0.83 0.84-0.89 ≥0.90	Women: ≤0.78 vs. ≥0.90
Li(2013) China	WC	Men: <78cm 78-82cm 83-86cm 87-91cm ≥92cm	Men: <78cm vs. ≥92cm
		Women: <70cm 70-74cm 75-79cm 80-84cm ≥85cm	Women: <70cm vs. ≥85cm
	WHR	Men: <0.85 0.85-0.88 0.89-0.90 0.91-0.94 ≥0.95	Men: <0.85 vs. ≥0.95
		Women: <0.77 0.77-0.78 0.79-0.81 0.82-0.84 ≥0.85	Women: <0.77 vs. ≥0.85
Keimling(2013) USA	WC	Men: <89.5cm 89.5-<93.5cm 93.5-<99.0cm 99.0-<106.5cm ≥106.5cm	Men: <89.5cm vs. ≥106.5cm
		Women: <73.6cm 73.6-<80.0cm 80.0-<86.0cm 86.0-<94.5cm ≥94.5cm	Women: <73.6cm vs. ≥94.5cm
	WHR	Men: <0.898 0.898-<0.929 0.929-<0.956 0.956-<1.000 ≥ 1.000	Men: <0.898 vs. ≥ 1.000
		Women: <0.746 0.746-<0.786 0.786-<0.826 0.826-<0.877 ≥ 0.877	Women: <0.746 vs. ≥ 0.877
Kabat(2015) USA	WC<br>WHR	ND	ND
Park(2011) UK	WC	Men: <88.0cm 88.0-93.0cm 93.1-97.6cm 97.7-103.2cm ≥103.3cm	Men: <88.0cm vs. ≥103.3cm
		Women: <73.0cm 73.0-78.0cm 78.1-83.3cm 83.4-90.4cm ≥90.5cm	Women: <73.0cm vs. ≥90.5cm
	WHR	Men: <0.883 0.883-0.915 0.916-0.944 0.945-0.978 ≥0.979	Men: <0.883 vs. ≥0.979
		Women: <0.739 0.739-0.772 0.773-0.803 0.804-0.843 ≥0.844	Women: <0.739 vs. ≥0.844
Folsom(2000) USA	WC	Women: <74.3cm 74.3-<80.0cm 80.0-<87.3cm 87.3cm-<96.0cm ≥96.0cm	Women: <74.3cm vs. ≥96.0cm
	WHR	Women: <0.762 0.762-<0.805 0.805-<0.848 0.848-<0.901cm ≥0.901	Women: <0.762 vs.≥0.901
Schoen(1999) USA	WC	Men: 69-91cm 91.1-97cm 97.1-104cm 104.1-145.5cm	Men: 69-91cm vs. 104.1-145.5cm
		Women: 32.5-82cm 82.1-91.5cm 91.6-101.1cm 101.2-167cm	Women: 32.5-82cm vs. 101.2-167cm
	WHR	Men: 0.61-0.93 0.931-0.97 0.971-1.00 1.01-2.33	Men: 0.61-0.93 vs. 1.01-2.33
		Women: 0.61-0.83 0.831-0.90 0.91-0.96 0.961-2.06	Women: 0.61-0.83 vs. 0.961-2.06
MacInnis(2006) Australia	WC	Men: <94cm 94-101.9cm ≥102cm	Men: <94cm vs.≥102cm
		Women: <80cm 80-87.9cm ≥88cm	Women: <80cm vs. ≥88cm
	WHR	Men: <0.90 0.90-0.94 ≥0.95	Men: <0.90 vs. ≥0.95
		Women: <0.75 0.75-0.79 ≥0.80	Women: <0.75 vs. ≥0.80
Ahmed(2006) USA	WC	Men: <102cm ≥102cm	Men: <102cm vs.≥102cm
		Women: <88cm ≥88cm	Women: <88cm vs. ≥88cm

WC, waist circumference; WHR, waist to hip ratio; ND, no data.

Figure S1. (a) Pooled relative risk of CC associated with waist circumference; (b) Pooled relative risk of CC associated with waist to hip ratio; (c) Pooled relative risk of RC associated with waist circumference; (d) Pooled relative risk of RC associated with waist to hip ratio

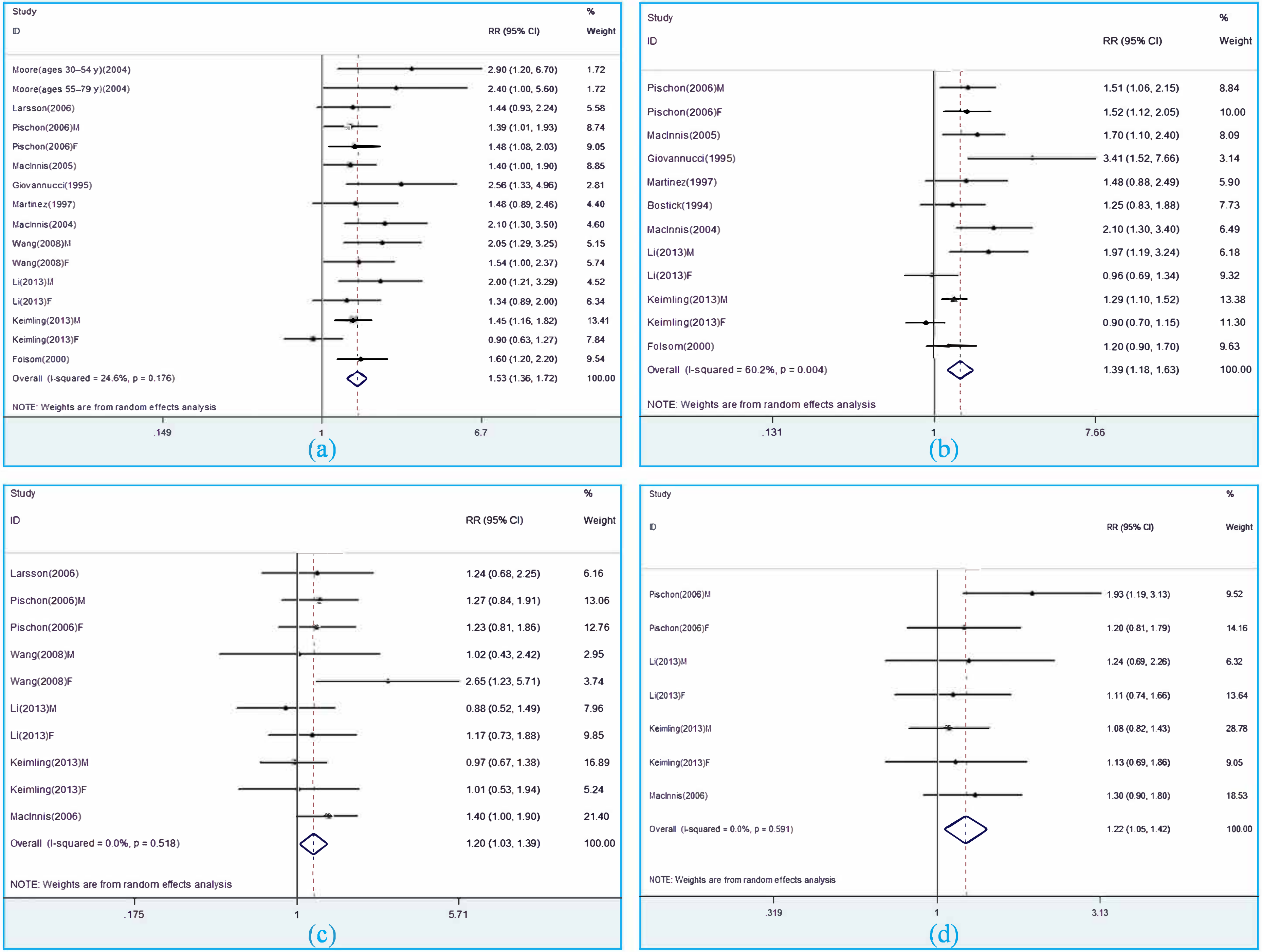

Study ID
RR (95% CI)
% Weight
Moore(ages 30–54 y)(2004) 2.90 (1.20, 6.70) 1.72
Moore(ages 55–79 y)(2004) 2.40 (1.00, 5.60) 1.72
Larsson(2006) 1.44 (0.93, 2.24) 5.58
Pischon(2006)M 1.39 (1.01, 1.93) 8.74
Pischon(2006)F 1.48 (1.08, 2.03) 9.05
MacInnis(2005) 1.40 (1.00, 1.90) 8.85
Giovannucci(1995) 2.56 (1.33, 4.96) 2.81
Martinez(1997) 1.48 (0.89, 2.46) 4.40
MacInnis(2004) 2.10 (1.30, 3.50) 4.60
Wang(2008)M 2.05 (1.29, 3.25) 5.15
Wang(2008)F 1.54 (1.00, 2.37) 5.74
Li(2013)M 2.00 (1.21, 3.29) 4.52
Li(2013)F 1.34 (0.89, 2.00) 6.34
Keimling(2013)M 1.45 (1.16, 1.82) 13.41
Keimling(2013)F 0.90 (0.63, 1.27) 7.84
Folsom(2000) 1.60 (1.20, 2.20) 9.54
Overall (I-squared = 24.6%, p = 0.176) 1.53 (1.36, 1.72) 100.00
NOTE: Weights are from random effects analysis
.149
1
6.7
(a)
Study ID
RR (95% CI)
% Weight
Pischon(2006)M 1.51 (1.06, 2.15) 8.84
Pischon(2006)F 1.52 (1.12, 2.05) 10.00
MacInnis(2005) 1.70 (1.10, 2.40) 8.09
Giovannucci(1995) 3.41 (1.52, 7.66) 3.14
Martinez(1997) 1.48 (0.88, 2.49) 5.90
Bostick(1994) 1.25 (0.83, 1.88) 7.73
MacInnis(2004) 2.10 (1.30, 3.40) 6.49
Li(2013)M 1.97 (1.19, 3.24) 6.18
Li(2013)F 0.96 (0.69, 1.34) 9.32
Keimling(2013)M 1.29 (1.10, 1.52) 13.38
Keimling(2013)F 0.90 (0.70, 1.15) 11.30
Folsom(2000) 1.20 (0.90, 1.70) 9.63
Overall (I-squared = 60.2%, p = 0.004) 1.39 (1.18, 1.63) 100.00
NOTE: Weights are from random effects analysis
.131
1
7.66
(b)
Study ID
RR (95% CI)
% Weight
Larsson(2006) 1.24 (0.68, 2.25) 6.16
Pischon(2006)M 1.27 (0.84, 1.91) 13.06
Pischon(2006)F 1.23 (0.81, 1.86) 12.76
Wang(2008)M 1.02 (0.43, 2.42) 2.95
Wang(2008)F 2.65 (1.23, 5.71) 3.74
Li(2013)M 0.88 (0.52, 1.49) 7.96
Li(2013)F 1.17 (0.73, 1.88) 9.85
Keimling(2013)M 0.97 (0.67, 1.38) 16.89
Keimling(2013)F 1.01 (0.53, 1.94) 5.24
MacInnis(2006) 1.40 (1.00, 1.90) 21.40
Overall (I-squared = 0.0%, p = 0.518) 1.20 (1.03, 1.39) 100.00
NOTE: Weights are from random effects analysis
.175
1
5.71
(c)
Study ID
RR (95% CI)
% Weight
Pischon(2006)M 1.93 (1.19, 3.13) 9.52
Pischon(2006)F 1.20 (0.81, 1.79) 14.16
Li(2013)M 1.24 (0.69, 2.26) 6.32
Li(2013)F 1.11 (0.74, 1.66) 13.64
Keimling(2013)M 1.08 (0.82, 1.43) 28.78
Keimling(2013)F 1.13 (0.69, 1.86) 9.05
MacInnis(2006) 1.30 (0.90, 1.80) 18.53
Overall (I-squared = 0.0%, p = 0.591) 1.22 (1.05, 1.42) 100.00
NOTE: Weights are from random effects analysis
.319
1
3.13
(d)
